# Dual-task walking and automaticity after Stroke: Insights from a secondary analysis and imaging sub-study of a randomised controlled trial

**DOI:** 10.1177/02692155211017360

**Published:** 2021-05-30

**Authors:** Johnny Collett, Melanie K Fleming, Daan Meester, Emad Al-Yahya, Derick T Wade, Andrea Dennis, Piergiorgio Salvan, Andrew Meaney, Janet Cockburn, Joanna Dawes, Heidi Johansen-Berg, Helen Dawes

**Affiliations:** 1 Centre for Movement, Occupational and Rehabilitation Sciences, Oxford Brookes University, Oxford, UK; 2 Wellcome Centre for Integrative Neuroimaging (WIN), FMRIB, Nuffield Department of Clinical Neurosciences, University of Oxford, UK; 3 School of Rehabilitation Science, The University of Jordan, Amman, Jordan; 5 Department of Health Sciences, Division of Physiotherapy, Brunel University, London, UK

**Keywords:** Stroke, gait, rehabilitation, dual-task interference, functional MRI

## Abstract

**Objective:**

To test the extent to which initial walking speed influences dual-task performance after walking intervention, hypothesising that slow walking speed affects automatic gait control, limiting executive resource availability.

**Design:**

A secondary analysis of a trial of dual-task (DT) and single-task (ST) walking interventions comparing those with *good* (walking speed ≥**0.8 m s** ^-1^ **, *n =* 21) and *limited* (walking speed** <**0.79 m s** ^-1^ **, *n* = 24) capacity at baseline.**

**Setting:**

Community.

**Subjects:**

Adults six-months post stroke with walking impairment.

**Interventions:**

Twenty sessions of 30 minutes treadmill walking over 10 weeks with (DT) or without (ST) cognitive distraction. *Good* and *limited* groups were formed regardless of intervention received. Main measures: A two-minute walk with (DT) and without (ST) a cognitive distraction assessed walking. *f*NIRS measured prefrontal cortex activation during treadmill walking with (DT) and without (ST) Stroop and planning tasks and an *f*MRI sub-study used ankle-dorsiflexion to simulate walking.

**Results:**

ST walking improved in both groups (Δbaseline: *Good =* 8.9 ± 13.4 m, *limited* = 5.3±8.9 m, Group × time = *P* < 0.151) but only the *good* walkers improved DT walking (△baseline: *Good =* 10.4 ± 13.9 m, *limited* = 1.3 ± 7.7 m, Group × time = *P* < 0.025). *f*NIRS indicated increased ispilesional prefrontal cortex activation during DT walking following intervention (*P* = 0.021). *f*MRI revealed greater DT cost activation for *limited* walkers, and increased resting state connectivity of contralesional M1 with cortical areas associated with conscious gait control at baseline. After the intervention, resting state connectivity between ipsilesional M1 and bilateral superior parietal lobe, involved in integrating sensory and motor signals, increased in the *good* walkers compared with *limited* walkers.

**Conclusion:**

In individual who walk slowly it may be difficult to improve dual-task walking ability.

**Registration:**

ISRCTN50586966

## Introduction

Enabling people after stroke to walk in the community remains a challenge.^1^ Community walking requires attention to navigate complex and changing environments, and after stroke people often fail to walk in such situations.^2^ The ability to attend to additional tasks whilst walking is impaired after stroke and leads to decrements in walking when a concurrent cognitive task (dual-task) is performed. To improve this ability interventions have incorporated dual-task walking training. Whilst, some improvements in dual-task walking speed have been found, the clinical significance of this approach is unclear^3^ and recent randomised controlled trials comparing dual and single-task walking intervention have not found dual-task training to be superior.^4,5^

Healthy walking is considered a largely automatic process with minimal use of executive resources. This ‘automaticity’ allows for walking to be controlled without continuous attentional monitoring and executive resources are available to attend to the environment and perform cognitive tasks.^6,7^ Proper consideration of walking automaticity may optimise recovery of dual-task related mobility limitations and community walking post stroke.

The cognitive processes engaged during walking can be inferred from observation of brain signals. For example, the amount of executive resource required to walk is reflected in prefrontal cortex activity.^8^ Functional Near-Infrared Spectroscopy (*f*NIRS) imaging has revealed increased Prefrontal cortex activation during walking in stroke survivors compared with healthy controls, substantiating the increased attentional demand required to achieve simple locomotion.^9^ When adding an additional task to walking this increased prefrontal cortex activity is exacerbated^9^ with studies generally finding prefrontal cortex activation increases with demands on attention.^6,8^

To elucidate *f*NIRS findings, Al-Yahya et al.^9^ used functional magnetic resonance imaging (*f*MRI), which has greater spatial resolution but is confined to simulated walking in the scanner. Greater activation of bilateral superior frontal gyrus, bilateral inferior temporal gyrus and left caudate nucleus was found in stroke patients during dual-task, relative to single-task conditions. They also report that increased activity under dualtask was related to task performance decrements. However, they specifically tested for *increased* activation during dual-tasking, whereas Burki et al.^10^ demonstrated decreases in activation of some motor regions (i.e. ‘dual-tasks savings’) in healthy young and older adults, rather than increases. This does seem to be dependent on both walking speed and executive function, raising the possibility that changes in brain activity during dual-tasking after stroke may depend on residual walking ability.

Walking, a highly evolved and automatic process, involves pathways and locomotor centres located throughout the central nervous system, driven by reciprocal afferent inputs produced by cyclic stepping.^6,7^ Therefore, the automatic processing and circuits involved in rhythmically controlling andco-ordinating walking may require a particular input configuration brought about by the pendula walking pattern.^7,11^ To achieve this, sufficient walking speed may be required in order to produce the required stimulus frequency to recover automatic processes in walking control.^12^ Indeed Dawes et al.^13^ found walking speed at entry predicted the response to body weight supported treadmill training after stroke. Furthermore the decrements brought about by dual-task walking are greater in stroke survivors with initially slower walking speeds.^14^ We hypothesise that Stroke survivors with slower walking speed may not have sufficient automaticity to ‘free up’ executive resources and therefore the capacity to improve dual-task walking, whereas those who can walk faster have greater executive resources available to improve this ability.

We used data from a randomised trial of dual and single-task walking interventions in stroke^4^ to compare mobility outcomes in those with good (walking speed ≥ 0.8 m s^-1^) and limited (walking speed <0.79 m s^-1^) capacity at baseline and used *f*NIRS and, in a sub study, *f*MRI to explore our automaticity hypothesis.

## Methods

### Design

This report is a secondary analysis of a single blinded two-arm parallel randomised controlled trial that compared single and dual-task treadmill walking training (1:1) in chronic stroke. Full details can be found in Meester et al.^4^ (Trial reg: ISRCTN50586966, National Research Ethics Service: 12/SC/0403). Here we performed a secondary analysis to compare those with good walking capacity with those with limited walking capacity^15^ at baseline. We utilised *f*NRIS and *f*MRI to test for difference in brain activation between good and limited walkers and response to walking training.

In absence of a cut off to define a minimal gait speed to drive automatic gait control a cut off speed of 0.8 m s^-1^ was used to define ‘good’ walking capacity. This speed has shown to be a good discriminator between limited and full community ambulation,^15^ which we assume is likely to require ‘sufficient’ automatic gait control. Using baseline two-minute walk test data, we formed two groups: Good (≥0.8 m s^-1^) and limited (<0.79 m s^-1^) walking capacity for comparison irrespective of which intervention they received.

### Participants

All participants provided informed consent and were recruited from the Thames valley, UK. Eligibility for this secondary analysis was the same as that for the main trail,^4^ briefly, the criteria were: (1) 18 years or older, (2) at least six months post any stroke, (3) had a reduced two-minute walk distance (slower than normative values for age^16,17^ or a visibly abnormal gait), (4) able to walk on a treadmill, (5) no concurrent neurological conditions or psychological disorder, (6) and no contraindication to safe participation in exercise. Recruited individuals were also invited to take part in the MRI sub study; to be eligible had to have had no contraindications to MRI.

### Intervention

Irrespective of intervention, 20 sessions were scheduled over 10 weeks in a quiet room with 1:1 supervision. Each session consisted of a 10 minutes warm-up, 30 minutes of walking in anaerobic zone (between 55% and 85% of the age predicted maximum heart rate (220 –age)) and 5 minutes cool down, all on a treadmill. Training was progressed to increase walking speed (and duration if 30 minutes could not be achieved).

Participant’s dual-task training had the addition of three types of distraction, each for 10 minutes, during the treadmill walking: (1) Discrete cognitive tasks, (2) a listening task and (3) Describing their plans for the day (see Supplement 1-Table 2 for training schedule).

### Assessment

This secondary analysis utilised data from assessments conducted at baseline and post training (scheduled within one week of completing the intervention). See Meester et al.^4^ and ISRCTN50586966 for all measures.

### Walking outcomes

Walking was assessed using a two-minute walk with and without the addition of a distracting task (recall of daily life activities, e.g. can you tell me how your day started?). Walking activity, average steps per day, was assessed over a week with a Step Watch Activity Monitor™ (OrthoCare Innovations, Seattle, WA). Perceptions of participation in community walking was assessed with the questions: ‘Do you get out of the house as much as you like?’ and ‘Do you feel confident when walking in the community?’ (‘yes’ or ‘no’ answer).

### Imaging-*f*NIRS

*f*NIRS (Oxymon, Artinis Medical Systems, The Netherlands) measured the pre-frontal cortex activation using a continuous wave (782, 859 nm). The eight optode configuration and data collection and processing can be found in Supplement 1.

*f*NIRS measurement was performed on the treadmill following a block design: Participants performed five tasks for 30 seconds alternated with 20 seconds rest (sitting on a chair on the treadmill). Tasks were presented in a random order to prevent the participant from anticipating the next task. Each task was repeated five times resulting in a total test-time of 21 minutes. The tasks were: Auditory Stroop task whilst standing, Walking at self-selected-walking-speed, Picture-planning task whilst standing, Auditory Stroop task whilst walking and the Picture-planning task whilst walking. Further details of the tasks and the block sequence can be found in Supplement 1.

### Imaging-*f*MRI

Magnetic Resonance Imaging (MRI) scanning was performed on a 3 Tesla Verio scanner (SIEMENS, Erlangen, Germany) using a 32-channel head coil. Initially a T1-weighted structural image was acquired followed by T2*-weighted Echo planar imaging sequences for the task- and resting state fMRI scans. Acquisition settings can be found in Supplement 1.

*f*MRI was acquired using the same block design as *f*NIRS except that a number Stroop task was used instead of the auditory Stroop and each task was only repeated four times. Responses were recorded via a rocker switch. During the rest period, the participant was instructed to keep still and focus on the word ‘Rest’ presented on the screen. Walking was simulated using a pedal task^18^ which involved alternating dorsi- and plantar-flexion, each foot in opposite phase at a self-selected frequency (see Supplement 1-Figure 3). All tasks were practiced beforehand.

### Analysis

This was a per-protocol analysis and participants who attended more than half their scheduled sessions were included. Baseline group comparisons for demographic and pre-intervention walking outcomes were performed using independent samples *T*-Test or Whitney *U* according to level of measurement and parametric assumptions.

Analysis of walking outcome comparing good and limited walkers groups was performed using the mixed linear model procedure in SPSS to determine within and between groups difference and group × time interactions. Analysis comparing the proportion of individuals in each group achieving minimal detectable change in two-minute walk distance (16 m improvement between test)^19^ was performed using the cross tabs procedure in SPSS and with the difference between groups determined by Pearsons *x* ^2^ and odds ratio with 95% CI.

*f*NRIS outcome analysis also used the mixed linear model procedure reported above comparing groups for ispi and contralesional activity during single and dual-task walking task.

Analysis of magnetic resonance imaging data obtained from the sub-study was performed using the FMRIB Software Library (FSL) (Version 5.0.8, FMRIB, Oxford UK^20^). Details of pre-processing steps and first level analyses can be found in Supplement 1. To enable group comparison, images of stroke survivors with left sided lesions were flipped so all lesions appeared in the right hemisphere.

For task *f*MRI at baseline the dual-task cost activation was compared between limited and good walkers in a higher-level analysis using FLAME.^21,22^ The correlation between the dualtask cost activation and two-minute dual-task walking distance was investigated by adding baseline dual-task two-minute walk distance as a covariate in a separate analysis. Resulting Z statistic images were thresholded with an initial cluster-forming threshold of Z = 3.1 followed by a family-wise error (FWE) corrected cluster extent threshold of *P* < 0.05. For longitudinal analysis, a fixed effects analysis was conducted at the individual subject level to contrast baseline with posttraining, followed by a mixed effects analysis using FLAME (Local Analysis of Mixed Effects) to compare between groups. Correlation with either baseline motor performance or the change in motor performance with training, was determined by adding dual-task two-minute walk or △dual-task two-minute walk as a covariate in separate analyses.

For resting state *f*MRI, a seed based analysis was conducted with primary motor cortex (M1) regions of interest. Group comparisons were conducted using the general linear model (independent samples *t*-test) and randomise, with 10,000 permutations.^23^ For longitudinal analysis, the connectivity map for each participant post-training was subtracted from baseline. The resulting image, depicting the change in connectivity, was compared between groups as above. A threshold-free cluster enhancement (TFCE) family-wise error rate corrected significance of *P* < 0.025 was used.

## Results

### Participants

Of the 50 people recruited to the main trial, 45 (*22 single-task and 23 dual-task training*) adhered to the interventions, resulting in 21 (*9 single-task, 12 dual-task training*) individuals stratified into the good and 24 (*13 single-task, 11 dual-task training*) in the limited walking capacity groups. Two individuals were lost to follow up, one from each intervention type and both limited walkers. Missing data due to poor signal quality for *f*NIRS was 31% and varied between channels and tasks (ranging from 14% to 48%). Sixteen individuals (*n =* 10 good, *n =* 6 limited walking capacity) were included in the *f*MRI sub study. Some participants were unable to complete the follow up scan and therefore the scans of five good walkers and five limited walkers scanned at both time points were used for longitudinal analysis.

Baseline demographic and pre-assessment data can be found in Table 1 (see Supplement 1 (Table 3) for sub-study particpants). Naturally, the good group and limited group differed across measures of walking capacity and Barthel index score was higher in the good walking group (*t* = -3.611, *P* = <0.001).

### Intervention

Adherence was similar across interventions (single-task: median = 20, Inter Quartile Range (IRQ) 16.5–20.0, dual-task: median = 20, IQR 19–20 sessions) and according to walking capacity group (Good:median = 20, IRQ 18.5–20.0, limited: 19.5, IQR 18–20 sessions).

### Mobility outcomes

There was an improvement in single-task two-minute walk distance (effect of time; *f* = 20.671, *P P* < 0.001) and no group × time interaction with (*f* = 2.137, *P* = 0.151), reflecting the finding that both groups improved after training (Good walker: *f* = 12.341, *P* = 0.002, limited walker: *f* = 8.265, *P*= 0.009). For dual-task, there was a significant group × time interaction (*f* = 5.399, *P* = 0.025), with only the good walkers improving post training (Good walker: *f* = 9.718, *P* = 0.005, limited walker: *f* = 0.672, *P* = 0.421) (Table 2).

Table 2 shows individual responses were consistent with these results, with no difference in the proportion of individuals achieving minimal detectable change in single-task two-minute walk distance (24% of good walkers, 13%, of limited walker: *P* = 0.172, OR: 0.753, 95%CI: 0.155–3.665) but significantly more good walkers achieving minimal detectable change in dual-task walking (33% of good walkers (*n* = 3 single-task, *n* = 5 training dual-task training), 5% of limited walkers (*n* = 1 dual-task training), *P* = 0.015 (OR:10.50, 95%CI: 1.16–94.93)). Table 2 also shows no significant difference between groups in perceptions of participation in community walking and neither group significantly increased their walking activity (steps per day).

These data suggested that while both groups showed comparable improvements in single-task walking following training, improvements in dualtask walking were associated with those with good walking capacity at baseline. We went on to investigate differences in brain signals between the limited and good walking groups.

### fNIRS

At baseline no significant difference in ipsilesional or contralesional prefrontal cortex activity were found between good and limited walkers for any tasks l (Table 1). Longitudinally a main effect of time was found for increased ispilesional prefrontal cortex activity during dual-task walking with the Stroop task (*f* = 6.152, *P* = 0.021), with no difference between groups (*f* = 0.168, *P* = 0.201) or in group × time interaction (*f* = 0.125, *P* = 0.723) (Table 2). No main effects were found contralesional prefrontal cortex activity. Task performance can be found in Supplement 1, correct responses to cognitive task ranged between 94 ± 8% and 78 ± 13%. There were no differences between groups or in group × time interaction in cognitive task performance (percentage correct responses or response time) during *f*NRIS measurement (see Supplement 1-Table 4).

### Task fMRI-baseline

Brain activation reflecting dual-task cost (vs single-tasks) was quantified separately for the planning task and for the Stroop task. For the planning task, at baseline a significantly greater dual-task cost activation was found in the contralesional hemisphere for the limited walkers in comparison with the good walkers (see Supplement 1 Figure 5.1, A). Areas of greater activation in limited walkers included contralesional precentral gyrus, superior and middle frontal gyrus and supplementary motor cortex. Supplement 1 Figure 5.1, B shows the percentage signal change for each participant and demonstrates that many of the good walkers show a decrease in activation during dual-tasks (dual-task saving). In comparison, the limited walkers tend towards increases in brain activation for the dual-task. The contralesional hemisphere dual-task cost activation for the planning task correlated negatively with dual task two-minute walk distance (see Supplement 1 Figure 5.2, A and C), such that participants with lower DT cost brain activity (or greater dual-task savings) walked further during dual-tasks tested outside the scanner.

For the Stroop task, there was no significant difference in dual-task cost activity between groups at baseline, but there was a significant negative correlation between dual-task cost activation and the dual-task vs walk distance (Z > 3.1, *P* < 0.05, see Supplement 1 Figure 5.2, B and D), such that participants with lower dual-task cost activity (or greater dual-task savings) walked further on the two-minute walk during dual-task tested outside the scanner. Areas of correlated activation include: bilateral precentral gyrus, middle frontal gyrus and paracingulate gyrus; contralesional superior parietal lobule, superior frontal gyrus and supplementary motor cortex; ipsilesional inferior frontal gyrus.

For the change in dual-task cost activation from baseline to post-intervention, there was no significant effect of group, nor were there any correlations between either baseline or change in dual-task two-minute walk and changes in dual-task cost activation with training.

### Resting state fMRI

For the ipsilesional M1 seed analysis, there were no significant voxel-wise differences between good and limited walkers at baseline. For the contralesional M1 seed analysis, at baseline there was significantly greater resting state connectivity between the contralesional M1 and several regions of the brain for the limited walkers in comparison with the good walking group (see Supplement 1 Figure 5.3). Regions showing greater connectivity include ipsilesional precentral gyrus, superior frontal gyrus and supplementary motor cortex.

For the longitudinal analysis, the change in connectivity was significantly greater for the limited group in comparison with the good walkers between the ipsilesional M1 seed and a small cluster in the ipsilesional precuneus and contralesional superior parietal lobe (see Supplement 1 Figure 5.4, A). Given that post-intervention connectivity maps were subtracted from baseline, (see Supplement 1 Figure 5.3, A) this indicates that the connectivity between the ipsilesional M1 and the ipsilesional precuneus/contralesional superior parietal lobe increases for good walkers, and decreases for the limited walkers.

There were no differences between groups for changes in connectivity between the contralesional M1 seed and other grey matter regions.

## Discussion

Our data supports that sufficient walking capacity (a walking speed of 0.8 m s^-1^ or greater used in this study) may be required to improve dual-task walking after stroke and improvement might not necessarily require specific dual-task walking training. These observations were elucidated by brain imaging data consistent with our automaticity hypothesis and may help to explain the unexpected lack of superiority of dual-task training over single task training when compared in randomised controlled trials.^4,5^

We found that both groups improved single-task walking speed but only the good walking capacity group improved dual-task walking. The overall treatment effect was small, consistent with the small treatment effect found in meta-analysis of physical exercise interventions on dual-task gait speed after stroke.^3^ Nevertheless, when considering individual response, we found a third of participants with good walking capacity achieved minimal detectable change in dual-task walking speed, three of whom received single-task training and five dual-task training. Our finding that improvement in dual-task walking speed was not dependent on receiving dual-task training is not necessarily surprising given the aforementioned meta-analysis^3^ found that only slightly larger treatment effects for dual-task walking after gait training incorporating additional tasks. Indeed our main trial analysis found no difference between single and dual tasking interventions, with both groups improving single task two-minute walk distance,^4^ with similar results also found recently by Plummer et al.^5^ While, a recent systematic review concluded that there were too few studies to support the hypothesis that participant’s initial walking capacity might contribute to the small treatment effects found for dual-task walking,^3^ walking capacity has previously been found to predict response to walking training in stroke survivors^13^ and this is further supported by our current analyses.

Despite the improvements in walking speed in both groups, and dual-task walking speed in the good walking capacity group, we found no improvement in walking activity or perception of community walking. Barclay et al.^24^ developed and verified a community ambulation model after stroke with perceptions of health and environmental factors impacting on an individual’s ability to ambulate in the community. Therefore, while walking speed and endurance are fundamental,^24^ multiple component interventions may be required improve community ambulation and participation in activities outside the home.^25^ Indeed stroke without leg weakness, or loss of coordination or visuospatial problems has been found to effect gait especially in participants with cognitive impairment.^26^

We used *f*NRIS to investigate the executive resource used during walking. Higher prefrontal cortex activity, as measured by *f*NIRS, has been observed across neurological conditions and in the elderly during single and dual-task walking.^8^ We did not find significant differences between our limited and good walking groups at baseline. While, the study may have been underpowered to detect differences, prioritising the motor task (in order to remain on the treadmill) may also contribute to this observation. Mori et al.^27^ found acceleration magnitude (a composite gait measure) negatively correlated with prefrontal cortex activation in stroke survivors, whereas prefrontal cortex activation correlated positively with cognitive task performance in the healthy group, inferring that stroke survivors prioritise motor tasks during dual-task conditions. Interestingly, in healthy individuals, treadmill walking has been to found to improve dual-task cognitive performance, compared to over ground walking, with the inference that the treadmill reduces the attentional cost of walking through cueing.^28^ This may be a further consideration in the of translation treadmill walking interventions to over ground walking performance. Following intervention (10 weeks for treadmill walking training) our data supports that more attention was dedicated to dual-tasking with an increase in ipsilesional prefrontal activity during walking with the Stroop task, irrespective of group. However, this was not reflected in improved cognitive task performance.

While cortical modulation is required to improve a complex behaviour like walking,^29^ *f*MRI has revealed neural correlates of improved automaticity in response to walking training in stroke through increased activation of subcortical networks and cortico-basalganglia–midbrain–cerebellar pathways.^29,30^ In the present study we proposed that those with limited walking capacity would have higher cortical activation to attend to the task and control of simulated walking. Indeed at baseline we found higher dual-task brain activation in contralesional frontal and motor areas in the limited walking group during the picture planning task interference and a negative correlation between dual-task activation and walking performance. This is consistent with a previous finding that increased contralesional activity is associated with worse gait function.^18^ Interestingly, our resting state network data revealed greater connectivity between the contralesional M1 and ipsilesional precentral gyrus, superior frontal gyrus and supplementary motor cortex in the limited walking group, brain areas which were found to have higher dualtask activation cost contralesionally in this group. These areas have been associated with conscious gait control using motor imagery in the scanner.^31^ Certainly supplementary motor cortex is believed to play a pivotal role in the cortical control by gait relaying sensory input and complex motor output^29,31^ and increased activation of the supplementary motor cortex is associated with worse affected leg functional impairment after stroke.^18^ Thus our data is consistent with a greater requirement for conscious gait control in the limited walking group.

In response to training we found resting state connectivity increased between the ipsilesional M1 and bilateral superior parietal lobe areas in good walkers, and decreased in limited walkers. The superior parietal lobes are central for the internal estimation of one’s self in relation to the surroundings by integrating sensory and motor signals.^32,33^ Specifically the increased connectivity with the precuneus in the good walking group might indicate adaption necessary for controlling gait to navigate complex environments. Indeed the precuneus has been found to have increased activity in healthy individuals over stroke patients during simulated walking^9^ and in older adults increased bilateral precuneus activity has found to be associated with better fast paced walking speed and obstacle navigation.^34^ Thus, whilst we did not find any improvement in community walking, these apparent brain adaptions are an encouraging observation.

There are a number of limitations that should be taken into account when considering our data and its interpretation. Firstly this was a secondary analysis and the study was not specifically designed or powered to test our hypotheses. Therefore the sample size was not sufficient for intervention type by walking ability comparison and necessitated good and limited walking groups be formed from individuals who had received different walking training interventions. Our choice of walking speed cut point was based on a discriminator of limited and full community ambulation after stroke,^15^ on the assumption this would reflect ‘sufficient’ automaticity. The balance between automatic and executive control of impaired walking is inevitably more complex than just walking speed^6^ and to our knowledge a minimal walking speed, when discrete steps requiring individual initiation become a reciprocal cycle, has yet to be determined. In choosing the 0.8 m s^-1^ our assumption was that to engage in full community ambulation a ‘sufficient’ level of automaticity would be required.

Poor signal quality in *f*NIRS measurement and the small sample size in the *f*MRI sub study, especially at follow up, makes these analyses particularly under powered. Nevertheless we found several, theoretically predicated, statistically significant insights and trends in the data. It should also be considered that the cognitive interference used during the two-minute walk test was different than that used during imaging assessments or walking on a treadmill and due to the unconstrained nature of the task performance was not formally evaluated. In addition, simulated walking in the scanner is not precisely analogous to over ground walking.^8,31^ However, this was necessary due to technological constraints and experimental paradigms^31^ and the combined use of these approaches provides complimentary data.^9^

In conclusion, this secondary analysis provides data to support the rationale that for those with limited walking capacity, initially increasing walking speed should be the priority. The data also supports that our automaticity hypothesis is worthy of further investigation and may help to explain the small dual-task treatment effects^3^ and lack of superiority of dual-task walking interventions.^4,5^ Greater understanding of this mechanism may help to better tailor intervention and direct a staged approach of increasing complexity for gait rehabilitation. Indeed we did not find any improvement in community walking outcomes supporting that multiple component interventions may be required to improve walking activities.^24^ Nevertheless, it is encouraging that the changes in brain activity that we found might be consistent with adaptions to support increased ability to navigate more complex environments.^34^

## Supplementary Material

Supplementary

## Figures and Tables

**Table 1:** Baseline group comparison

Demographics and descriptors	Good walker	Limited walker	*P* Value
*N*	21	24	
Age (years)	62 ± 14	62 ± 14	0.878
Sex (m:f)	13:8	11:13	0.281
Stroke type (isc:hem:both)	16:5:0	11:11:2	0.082
Stroke location (r:l:mid)	10:8:3	12:8:4	0.939
Time since stroke (months)	51 ± 59	32 ± 43	0.255
Barthel index	20 (20–20)	19 (18–20)	<0.001
MOCA	26 (24–28)	26 (22–28)	0.639
Walking behavioural			
Support (none:cane:person)	15:6:0	2*:18*:4*	<0.001
2 minutes single task (m)	120.7 ± 20.0	56.9 ± 17.0	<0.001
2 minutes dual task (m)	103.1 ± 20.2	51.4 ± 16.0	<0.001
Average steps per day	4423 ± 1368	2078 ± 858	<0.001
Out of the house as much as you like? (y:n)	17:4 (yes: 81%)	10:13 (yes: 43%)	0.011
Confident walking in the community (y:n)	16:5 (yes: 76%)	10:13 (yes: 43%)	0.027
Walking fNIRS			
Walking Ips PFC (OHb mmol/L)	0.67 ± 0.26 (*n* = 13)	0.80 ± 0.19 (*n* = 17)	0.136
Walking con PFC (OHb mmol/L)	0.64 ± 0.30 (*n* = 15)	0.72 ± 0.28 (*n* = 16)	0.435
DT (stroop) Ips PFC (OHb mmol/L)	0.33 ± 0.38 (*n* = 13)	0.22 ± 0.39 (*n* = 16)	0.425
DT (stroop) Con PFC (OHb mmol/L)	0.49 ± 0.32 (*n* = 15)	0.24 ± 0.40 (*n* = 15)	0.066
DT (planning) Ips PFC (OHb mmol/L)	0.42 ± 0.36 (*n* = 13)	0.37 ± 0.31 (*n* = 17)	0.687
DT (planning) Con PFC (OHb mmol/L)	0.34 ± 0.39 (*n* = 15)	0.30 ± 0.22 (*n* = 15)	0.752

Descriptive statistic report Mean ± Standard deviation, Median (Interquartile range) or ratio xx:xx.

MOCA: montreal cognitive assessment; isc: ischemic; hem: haemorrhagic; Y: yes; n: no: DT: dual-task; ST: single-task; PFC: prefrontal cortex; Ips: ispilesional; con: contralesional.

* Denotes significant different proportions between groups for subsets

*P* = probability value from independent samples *t* test, Mann–Whitney U or Persons Chi Squared of comparison between Good and limited walkers.

**Table 2:** Longitudinal analysis

Walking behavioural	Good walker		Limited walker		Between group comparison
	Change form baseline	Exploratory within group	Change from baseline	Exploratory within group	
ST 2-minute walk distance (m)	8.9 ± 13.4	*P* = 0.002	5.3 ± 8.9	*P* = 0.009	Group: P = <0.001 Group × time Interaction: *P* = 0. 151
DT 2-minute walk distance (m)	10.4 ± 13.9	*P* = 0.005	1.3 ± 7.7	*P* = 0.421	Group: P = <0.001 Group × time Interaction: *P* = 0.025
ST MDC *(n)* [%]	5 [23.8]		3 [12.5]		Group: *P* = 0. 172 OR: 1.98, 95%CI: 0.41-9.6
DT MDC *(n)* [%]	7 [33.3]		1 [4.5]		Group: *P* = 0.015 OR: 10.50, 95%CI: 1.16-94.93
Average steps per day	220 ± 1270	*P* = 0.499	-96 ± 820	*P* = 0.731	Group: P < 0.001 Group × time Interaction: *P* = 0.423
Out of the house as much as you like? (yes)	-1	*P* = 0.707	0	*P* = 1.000	Group: *P* = 0.725 OR: 0.753, 95%CI: 0.155-3.665
Confident walking in the community (yes)	3	*P* = 0.214	-2	*P* = 0.175	Group: *P* = 0.732 OR: 1.305, 95% CI 0.285-5.960
FNIRS					
Walking Ips PFC (OHb mmol/L)	0.02 ± 0.31		-0.19 ± 0.43		Group: *P* = 0.479 Group × time Interaction: *P* = 0. 189
Walking con PFC (OHb mmol/L)	-0.16 ± 0.49		-0.01 ± 0.17		Group: *P* = 0.290 Group × time Interaction: *P* = 0. 130
DT (stroop) Ips PFC (OHb mmol/L)	0.18 ± 0.18		0.25 ± 0.55		Group: *P* = 0.201 Group × time Interaction: *P* = 0.723
DT (stroop) Con PFC (OHb mmol/L)	-0.11 ± 0.53		0.30 ± 0.33		Group: *P* = 0. 125 Group × time Interaction: *P* = 0.061
DT (planning) Ips PFC (OHb mmol/L)	0.03 ± 0.42		0.23 ± 0.27		Group: *P* = 0.578 Group × time Interaction: *P* = 0.202
DT (planning) Con PFC (OHb mmol/L)	0.04 ± 0.30		0.16 ± 0.21		Group: *P* = 0.875 Group × time Interaction: *P* = 0.320

Descriptive statistic report as change from baseline (post training - baseline): Mean ± Standard deviation, *n =* number of people in group achieving MDC (minimal detectible change (increase)) in 2-minute walk distance ([%] = within group percentage achieving MDC), yes = number of people in changed answered from no to yes to the question. P value for exploratory within group inferential statistics reported in column ‘Exploratory within group’ following group descriptive statistics. Main analysis of group comparisons ‘Between Group comparison’ reports between group difference (Group) and group time interaction (Group × time Interaction) or between groups.

OR: Odds Ratio; CI: 95% confidence intervals; DT: dual task; ST: single task; PFC: prefrontal cortex; Ips: ipsilesiona; Con: contralesional; m, metres; OHbmmol.L: oxyhaemoglobin mmol per litre.

## Clinical message

- After intervention single-task walking improved in both limited and good walkers but only good walkers improved dualtask walking.
- This finding, supported by MRI data, is consistent with the hypothesis that those who walk slowly have limited automatic gait control and reduced capacity to improve dual-task walking.
