# Supplementary material for: Dual-task walking and automaticity after Stroke: Insights from a secondary analysis and imaging sub-study of a randomised controlled trial
Source: Clin Rehabil. Author manuscript; Available in PMC 2021 Nov 1. (PMC8524683; doi:10.1177/02692155211017360)
Supplement: Supplementary [file EMS129487-supplement-Supplementary.pdf]

Supplement 1.

Supplementary Methods

Setting

Assessments took place at the movement science laboratory, Oxford Brookes University (clinical and *f*NIRS assessment) and Wellcome Centre for Integrative Neuroimaging (FMIRB), University of Oxford (MRI assessment). The Intervention took place at either Clinical Exercise and rehabilitation Unit, Oxford Brookes University or St Crispin's Leisure Centre, Wokingham.

Walking outcomes:

Walking function

The 2 minute walk tests were performed on a 16 meter track set out with two cones on a corridor (not in use during the test). Instructions were to walk at their normal walking speed, make turns around the cones and to continue walking until asked to stop and the distance covered measured. One 2 minute walk was performed in which the participants walked free from distraction. A second test was performed where participants were asked questions whilst performing the test (dual task). Questions focussed on the person's planning of daily activities (e.g. "Can you tell me how your day started today?"). The order of the test was random to account for differences in walking distance between the two tests as a result of tiredness during the second walk test.

Walking activity

Data gathered with the StepWatch Activity Monitor was extracted and processed with StepWatch™ Analysis Software 3.1. (OrthoCare Innovations, Seattle). Monitors were worn for 7 full days and data from those 7 days was analysed. During the study, some participants indicated that they had worn monitors the wrong way around or forgot to put on in the morning, therefore it was decided that days showing a total amount of step counts less than 10% of the daily average across a week were disregarded and considered as monitor not (correctly) worn. For all other days, up to a maximum of 7 days, average steps per day, maximum daily step count per week and sum of all steps taken in a week were calculated by the device software.

***f*NRIS data acquisition and processing:**

Data was collected and processed using system software (Oxysoft 2.1.6, Artinis Medical Systems, The Netherlands). Light intensity changes (modified Beer-Lambert law) sampled at 10Hz to calculate

relative changes in HHb and OHb concentrations. A low pass filter of 0.7Hz was used to remove high frequency noise and enable visual inspection of signals for motion artefacts. Blocks of data containing motion artefacts, missing signals or other noise were removed from analyses. Traces were then filtered with a moving Gaussian filter (1), using a width of 4s. Blocks included after motion artefact analysis and filter processing were detrended for the first 5 seconds preceding the task start and averaged for the task period and 20-second rest period after each task. For between subjects comparison, average signals for task and rest were normalized by dividing the whole average trace by the maximum concentration change within a channel and across tasks (2). The average relative concentration changes were calculated for the middle 10 seconds of both task and rest periods and used for statistical analyses.

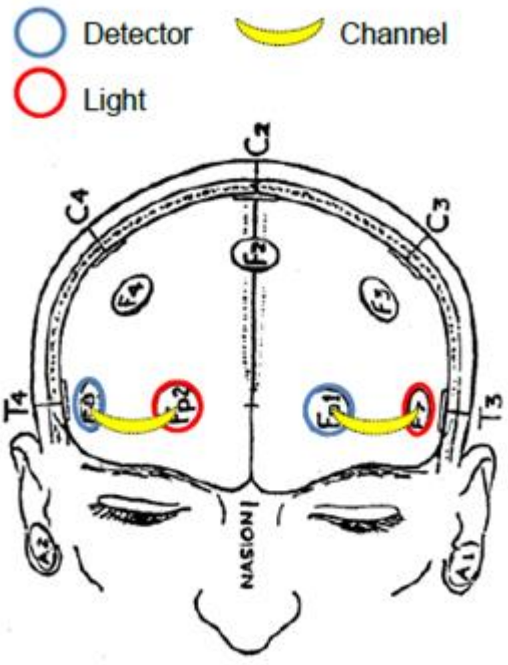

Sup Figure 1 Anterior view of Prefrontal *f*NIRS optode placement.

Two detectors and light sources were used to create two channels which were placed over the left and right PFC covering an area between F7 and Fp1 and F8 and Fp2. Inter-optode-distance was 30mm.

Treadmill walking: Self-selected speed (fNIRS)

To obtain selected walking speed the researcher exposed the stroke survivor to a range of speeds on the treadmill without giving feedback about the speed. The participants then chose the speed which they found comfortable and practised walking at this speed to confirm its selection and get familiarised to treadmill walking. If needed the person was allowed to hold one or both side-bars of

the treadmill whilst standing and walking. In some cases participants received some extra familiarisation sessions outside the assessment to get comfortable with treadmill walking.

Sup table 1 'Fixed Random' order tasks during of *f*NRIS measurement

Time	Task	Instruction	Event
0:20		Start Audio task	A
1:08		Start Walking	B
2:01		Start Picture task	C
2:49	+	Start Walking and Audio task	D
3:37		Start Walking	B
4:30	+	Start Walking and Picture task	E
5:18		Start Picture task	C
6:06	+	Start Walking and Audio task	D
6:57		Start Audio task	A
7:47		Start Walking	B
8:38	+	Start Walking and Picture task	E
9:26		Start Picture task	C
10:14	+	Start Walking and Audio task	D
11:05	+	Start Walking and Picture task	E
11:53		Start Audio task	A
12:44		Start Picture task	C
13:29		Start Walking	B
14:22	+	Start Walking and Audio task	D
15:13	+	Start Walking and Picture task	E
16:01		Start Audio task	A
16:52		Start Audio task	A
17:43		Start Picture task	C
18:28		Start Walking	B
19:21	+	Start Walking and Audio task	D
20:12	+	Start Walking and Picture task	E
21:00	END	END	

MRI data acquisition and processing

Structural image

Acquisition settings: Flip angle = 8°, TR = 2040ms, TE = 4.7ms, matrix size = 192 x 192, FOV = 192mm, slice thickness 3.0mm, voxel size = 1.0 x 1.0 x 1.0mm.

Processing: Brain extraction of the T1-weighted image was performed using OptiBET (Optimized brain extraction for patient brain) (3). The lesion was manually masked on the T1-weighted image using FSLview. Images were transformed to standard space (MNI 152), excluding the lesion region, using FMRIB's registration tools FLIRT (4) and FNIRT (5).

Task-fMRI

Acquisition settings: flip angle = 90°, TR = 2000ms, TE = 30ms, matrix size = 64 x 64, FOV = 192mm, slice thickness 3.0mm, voxel size = 3.0 x 3.0 x 3.0mm and 36 slices per volume. 512 measurements were taken during the scan with partial coverage of the cerebellum.

Processing: Initially, independent component analysis (ICA) performed by FSL's Multivariate Exploratory Linear Optimized Decomposition into Independent Components (MELODIC) was used to split the data into artefactual and non-artefactual components. Two trained researchers (DM & AD) individually went through the MELODIC output and identified components showing artefacts based on temporal and spatial features, following published protocols (6) . Noise components were removed from the fMRI data, then the de-noised datasets were entered into FEAT (7) for further analysis. Pre-processing included motion correction using MCFLIRT (8), non-brain removal using BET (9), spatial smoothing using a Gaussian kernel of 5 mm FWHM, grand-mean intensity normalisation and highpass filtering.

Statistical procedures were carried out in FEAT, with FMRIB's Improved Linear Model (FILM) (7). A boxcar regressor modelling the task and rest blocks was used to create first-level statistical maps, with each task modelled as a separate explanatory variable, for each participant at baseline and post-training separately. The "dual task cost" was determined by contrasting brain activation during dual task conditions with the sum of the activation during each of the corresponding single task conditions. For higher level analysis, EPI images were co-registered to the bias-corrected T1-weighted image and then to MNI space using FLIRT.

Resting state

Acquisition settings: flip angle = 90°, TR = 3000ms, TE = 30ms, matrix size = 64 x 64, FOV = 192mm, slice thickness 3.0mm, voxel size = 3.0 x 3.0 x 3.0mm and 54 slices per volume. 120 measurements were taken during the scan with partial coverage of the cerebellum.

Processing: EPI images were pre-processed using MELODIC, including motion correction using MCFLIRT (8), spatial smoothing using a 6 mm FWHM Gaussian kernel and temporal filtering. Noise components were classified manually (MF), according to published criteria (10) and removed using

FSL regfilt. The resulting de-noised images were co-registered to the bias-corrected T1-weighted image then transformed to MNI space, using FLIRT (8) and FNIRT (5) for further analysis.

Seed based connectivity analysis was conducted with primary motor cortex (M1) regions of interest (ROI), using the human motor area template (11). Dual regression was used to extract the timeseries from the ipsilesional and contralesional M1 ROI (in separate analyses) and correlate the timeseries with that of each voxel of the brain.

Pedal task (fMRI)

The pedal task required participants pedal (alternating dorsi- and plantar-flexion), each foot in opposite phase at a self-selected frequency on a purpose-built apparatus whilst lying on their back in the scanner (sup fig 2). An image of a moving foot was presented on the left side of the screen and participants were asked to pedal until the foot-image disappeared from the screen (sup fig 3)

A **B**

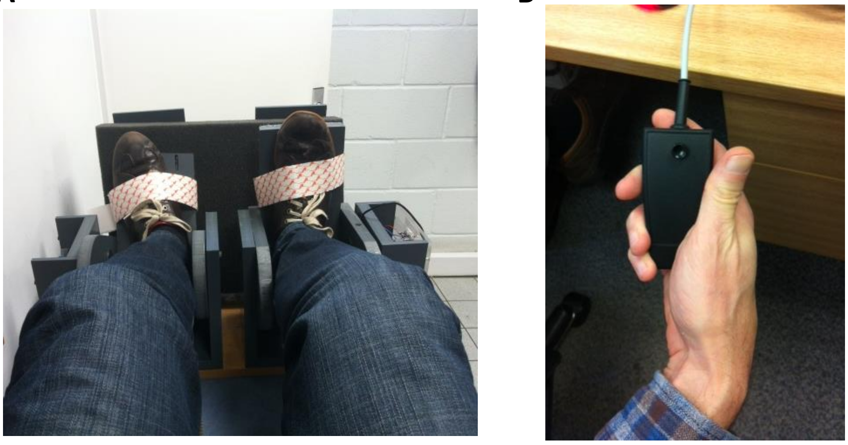

Sup Figure 2 Instruments used for the task-fMRI scan.

A: Bipedal device. The left and right pedals move independently of each other. **B:** Rocker switch used to indicate the participant’s response on the cognitive (Number stroop and picture planning) tasks.

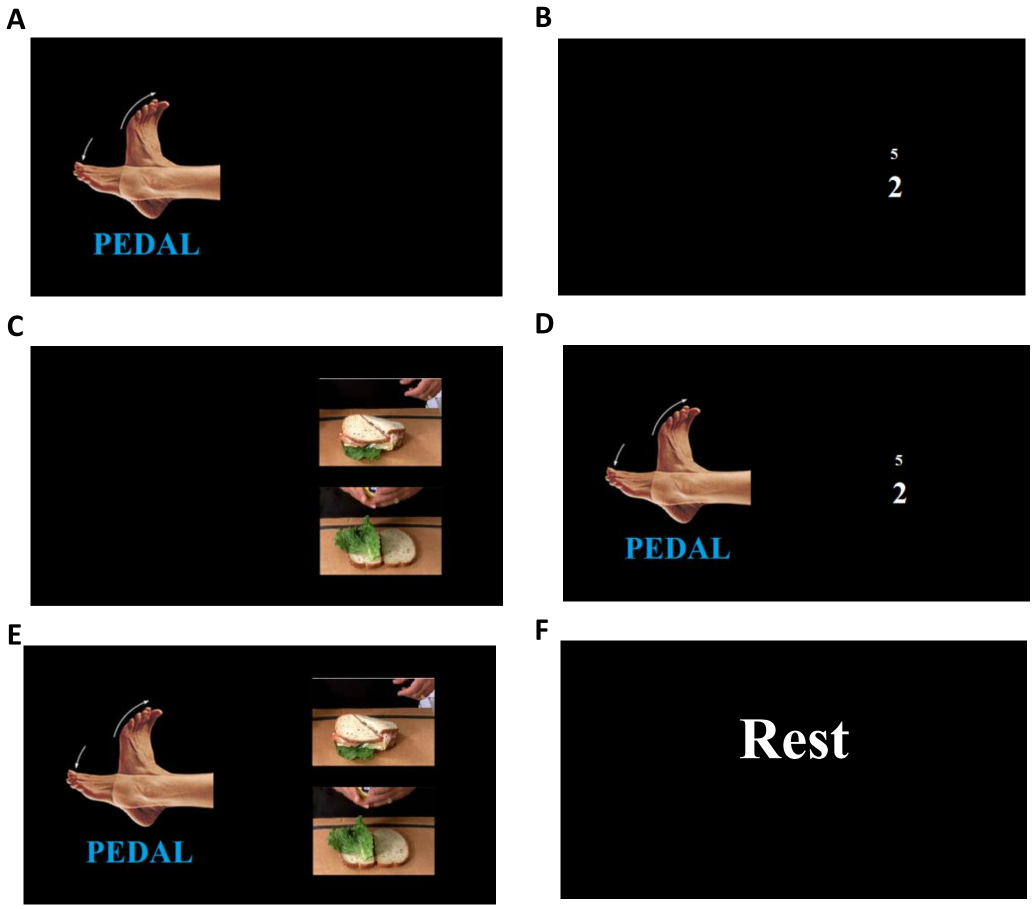

Sup Figure 3. Stimuli presented during Task-fMRI

Stimuli presented during the task-fMRI: A. pedalling alone, B. number stroop task alone (NS), C picture-planning task alone (PP), D number stroop whilst pedalling task (NS-DT), E. picture-planning whilst pedalling task (PP-DT), and F. rest blocks

Cognitive tasks

Picture planning (fNIRS and fMRI)

Picture planning task was a customised task, designed to test executive function in a manner that simulates distractions during community walking. Two pictures of the same daily activity were presented on the screen, and each picture represented a different action within the activity. The objective was to indicate whether the top or bottom picture came first in the sequence (sup fig 4). During *f*NIRS indication was verbal and *f*MRI used a rocker switch (sup fig 2)

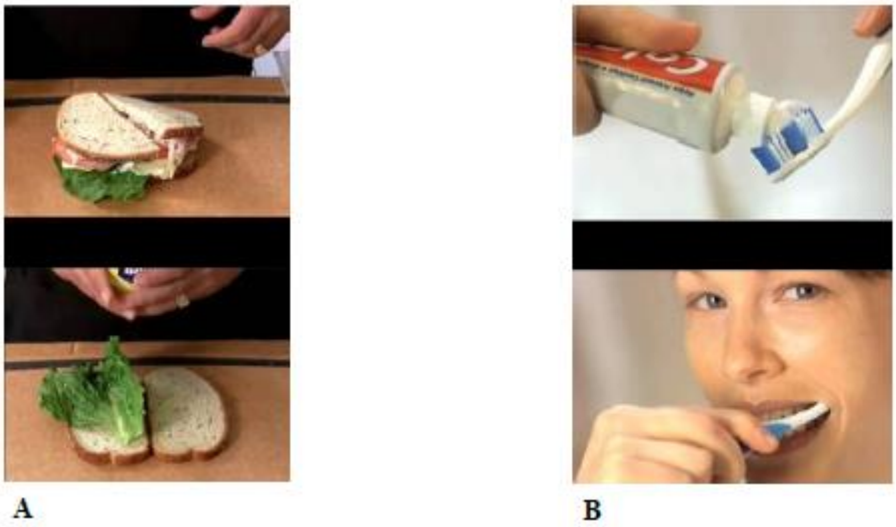

Sup Figure 4. Two examples of stimuli represented during the picture planning task.

The objective of the task was to verbally indicate the first picture in the sequence. A. Two pictures of a person who is making a sandwich, the bottom picture is the first picture in the sequence. B. Two pictures of a woman who brushes her teeth, the top picture is the first picture in the sequence.

Auditory Stroop (fNIRS)

The auditory variant of the Stroop task (12) required the participant to listen to the words "High" and "Low" spoken out at either a high pitch or a low pitch. The participant was asked to verbally indicate, by saying "high" or "low", whether the word was spoken out at a high or low pitch. The inter-stimulus-interval was set at 3.5s

The Number Stroop (fMRI)

The number Stroop variant of the Stroop task used visual stimuli presented on the screen; each stimulus consisted of a set of two numbers which were presented for 2 seconds. The numbers were presented vertically, one above the other, and differed in magnitude. In addition, the two numbers differed in font size. The objective was to indicate whether the top number or the bottom number was the biggest number in magnitude, irrespective of font size, using the rocker switch.

Intervention

Dual-task intervention schedule can be found in Supplementary table 2

Sup table 2. Dual-task intervention session schedule

Task	Description	Duration
	**Cognitive Task Block**	2 * 5min
Auditory Stroop	*A randomized series of the word "High" and "Low" are played through speakers at a high or low pitch. The subject must state the pitch of the word that was just said.*	2min30sec
Serial Subtraction	*The person is asked to count backwards from a number between 290 and 300 in steps of 3, 4 or 7.*	2min30sec
Clock Face Task	*A time is given verbally and the person must state whether the corresponding clock face has hands on the left, right or both sides of the clock.*	2min30sec
Letter Fluency	*A letter of the alphabet is given and the person is asked to name as many words as they can think of that start with that letter.*	2min30sec
Alternative uses	*The person is given an object and has to come up with alternative uses for that object.*	2min30sec
Creativity	*The purpose of the task is to name as many objects that have a certain attribute (e.g. objects that are tall).*	2min30sec
	**Radio**	10min
Radio or other audio fragment	*An audio fragment is played which is then used as topic of conversation between the trainer and trained person.*	10min
	**Planning**	2 * 5min
Planning of activities of daily living	*The person is asked to describe how they plan their daily activities; from short actions as: making a cup of tea, to planning an upcoming day out or holiday.*	5min

Supplementary Results

Sup table 3. *f*MRI Sub study baseline descriptive data and pre-intervention walking data

Demographics and descriptors	Good walker	Limited walker	*p*
N	*10*	*6*	*Na*
Age (years)	*61±10*	*69±9*	*0.124*
Sex (m:f)	*6:4*	*2:4*	*0.302*
Stroke type (isc:hem:both)	*10:0:0*	*6:0:0*	*Na*
Stroke Location (r:l:mid)	*5:3:2*	*3:5:0*	*0.449*
Time Since Stroke (months)	*44±45*	*47±76*	*0.936*
Barthel Index	*20 (20-20)*	*20 (18-20)*	*0.428*
MOCA	*27 (24-28)*	*23 (18-28)*	*0.093*
Walking behavioural			
Support (none:cane:person)	*7:3:0*	1:5:0	*0.039*
2min Single task (m)	*112.2±19.3*	*57.6±16.1*	*>0.001*
2min Dual Task (m)	*107.2±*17.6	*51.9±13.4*	*>0.001*

Mean±Standard deviation, Median (Interquartile range), *p* = probability value from independent samples t test, man Whitney U or Persons Chi Squared of comparison between Good and limited. Abbreviations: MOCA (Montreal Cognitive Assessment)

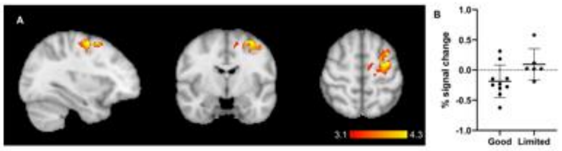

Figure 5.1

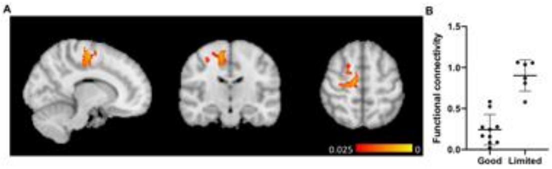

Figure 5.3

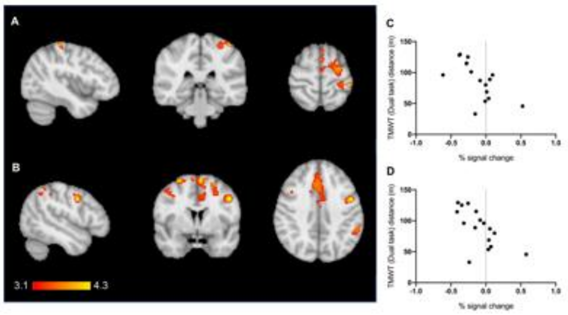

Figure 5.2

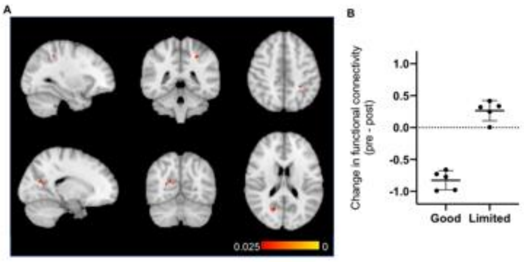

Figure 5.4

Sup Figure 5. fMRI Outcomes.

Figure 5.1 Greater dual task cost activation for the limited walking group in comparison with the good walking group. A: Group level contrast limited > good walkers for dual task cost (PP + Pedal − (PP alone + pedal alone) at baseline. Clusters determined by Z > 3.1 and a FWE corrected cluster significance threshold of P < 0.05. The contralesional hemisphere is shown on the right-hand side of the image. Slices centred on cluster maxima. B: Percent signal change within the significant clusters for individual participants (with mean and standard deviation indicated). **Figure 5.2** Areas of dual task cost activation correlate negatively with dual task motor performance (TMWT-DT distance). Whole group (n = 16) correlation between dual task cost activity and dual task TMWT distance at baseline A, C: PP + Pedal − (PP alone + pedal alone). B, D: NS + Pedal − (NS alone + pedal alone). Clusters determined by Z > 3.1 and a FWE corrected cluster extent significance threshold of P < 0.05. The contralesional hemisphere is shown on the right-hand side of the image. Slices centred on cluster maxima. The scatterplots (C, D) show the percent signal change for individual participants from within the significant clusters. **Figure 5.3**. Greater resting state connectivity with contralesional M1 for the limited walkers at baseline. A: Group level contrast limited > good walkers for connectivity with contralesional M1 seed (TFCE-corrected P < 0.025). The contralesional hemisphere is shown on the right-hand side of the image. Slices centred on cluster maxima. Colour bar indicates P value range. B: Functional connectivity values from within the significant clusters for each participant (with group mean and standard deviation indicated). **Figure 5.4.** Greater change in resting state connectivity with ipsilesional M1 (pre-post intervention) for the limited walkers. A: Group level contrast limited > good walkers for post-intervention connectivity (ipsilesional M1 seed) subtracted from baseline (FWE corrected P < 0.025). The contralesional hemisphere is shown on the right-hand side of the image. Slices centred to display the significant clusters. Colour bar indicates P value range. B: Functional connectivity change values from within the significant clusters for each participant (with group mean and standard deviation indicated). Negative values indicate greater functional connectivity post-intervention compared with baseline.

Sup table 4. Cognitive performance during dual-task *f*NIRS measurement

Dual task cognitive performance	Good walker		Limited walker		Between Group comparison
Task and performance measure	baseline	Post training	baseline	Post training	
Stroop % correct responses	92%±11%	89%±22%	94%±8%	91%±11%	Group: p=0.603<br>Group*time Interaction: p=0.972
Stroop response time (sec)	1.41±0.21	1.37±0.22	1.39±0.33	1.37±0.24	Group: p=0.290<br>Group*time Interaction: p=0.130
Planning % correct responses	83%±11%	83%±9%	79%±14%	78%±13%	Group: p=0.153<br>Group*time Interaction: p=0.959
Planning response time (sec)	1.91±0.41	1.95±0.34	2.04±0.41	2.02±0.55	Group: p=0.443<br>Group*time Interaction: p=0.570

Mean±standard deviation of performance (% of Correct reponses to the stimuli and the time taken to respond to the stimuli) on cognitive tasks during treadmill walking as part of the *f*NRIS block design. Task were presented on a computer monitor using 'Presentation'® software (Presentation version 16.5). Participants verbal responses were recording using this software that calculated the response times (time between stimuli and verbal response).

Supplementary References

1. Molavi, B. and G. A. Dumont (2012). "Wavelet-based motion artifact removal for functional near-infrared spectroscopy." Physiol Meas 33(2): 259-270.
2. Koenraadt, K. L., E. G. Roelofsen, J. Duysens and N. L. Keijsers (2014). "Cortical control of normal gait and precision stepping: an fNIRS study." Neuroimage 85 Pt 1: 415-422.
3. Lutkenhoff E.S, M Rosenburg, J Chiang, et al (2014) "Optimized Brain Extraction for Pathological Brains (optiBET)."Plos one https://doi.org/10.1371/journal.pone.0115551
4. Jenkinson M and Smith S. (2001) "A global optimisation method for robust affine registration of brain images" Med. Image Anal. 5: 143-156
5. Andersson J., Jenkinson M., Smith. S. (2007) "Non-linear Registration, aka Spatial Normalisation" FMRIB Technical Report TR07JA1
6. Salimi-Khorshidi, G., G. Douaud, C. F. Beckmann, M. F. Glasser, L. Griffanti and S. M. Smith (2014). "Automatic Denoising of Functional MRI Data: Combining Independent Component Analysis and Hierarchical Fusion of Classifiers." Neuroimage 90: 449-468.
7. Woolrich, M. W., T. E. Behrens and S. M. Smith (2004). "Constrained linear basis sets for HRF modelling using Variational Bayes." Neuroimage" 21(4): 1748-1761.
8. Jenkinson, M., P. Bannister, M. Brady and S. Smith (2002). "Improved optimization for the robust and accurate linear registration and motion correction of brain images." Neuroimage 17(2): 825-841.
9. Smith, S. M. (2002). "Fast robust automated brain extraction." Hum Brain Mapp 17(3): 143-155.

10. Griffanti L, Douaud G, Bijsterbosch J (2017) “Hand classification of fMRI ICA noise components” NeuroImage 154: 188-205.
11. Mayka M, Corcos, DM (2006) Three-dimensional locations and boundaries of motor and premotor cortices as defined by functional brain imaging: a meta-analysis” NeuroImage 31(4):1453-74.
12. R E Shor “An auditory analog of the Stroop Test” (1975) J Gen Psychol 93:281-8.
